# A Single Vaccination with an Improved Nonspreading Rift Valley Fever Virus Vaccine Provides Sterile Immunity in Lambs

**DOI:** 10.1371/journal.pone.0077461

**Published:** 2013-10-22

**Authors:** Nadia Oreshkova, Lucien van Keulen, Jet Kant, Rob J. M. Moormann, Jeroen Kortekaas

**Affiliations:** 1 Department of Virology, Central Veterinary Institute, part of Wageningen University and Research Centre, Lelystad, The Netherlands; 2 Department of Infectious Diseases and Immunology, Virology Division, Faculty of Veterinary Medicine, Utrecht University, Utrecht, The Netherlands; George Mason University, United States of America

## Abstract

Rift Valley fever virus (RVFV) is an important pathogen that affects ruminants and humans. Recently we developed a vaccine based on nonspreading RVFV (NSR) and showed that a single vaccination with this vaccine protects lambs from viremia and clinical signs. However, low levels of viral RNA were detected in the blood of vaccinated lambs shortly after challenge infection. These low levels of virus, when present in a pregnant ewe, could potentially infect the highly susceptible fetus. We therefore aimed to further improve the efficacy of the NSR vaccine. Here we report the expression of Gn, the major immunogenic protein of the virus, from the NSR genome. The resulting NSR-Gn vaccine was shown to elicit superior CD8 and CD4-restricted memory responses and improved virus neutralization titers in mice. A dose titration study in lambs revealed that the highest vaccination dose of 10^6.3^ TCID_50_/ml protected all lambs from clinical signs and viremia. The lambs developed neutralizing antibodies within three weeks after vaccination and no anamnestic responses were observed following challenge. The combined results suggest that sterile immunity was achieved by a single vaccination with the NSR-Gn vaccine.

## Introduction

Rift Valley fever virus (RVFV) is a mosquito-transmitted pathogen that infects domesticated ruminants as well as humans. The virus circulates on the African continent and caused several outbreaks in countries outside the African mainland [1]–[5]. In ruminants, RVFV causes massive abortion storms and high mortality among young animals [6]–[8]. In humans the infection manifests mainly as a acute, self-limiting febrile illness which may involve headache, malaise, myalgia, arthralgia and gastro-intestinal symptoms. Humans can however develop complications, which include retinal damage, jaundice, neurological disease and haemorrhagic fever [9], [10]. The case fatality rate in humans is historically reported to be between 0.5 and 2% [11].

RVFV belongs to the *Phlebovirus* genus of the family *Bunyaviridae*. Typical for a bunyavirus family member, RVFV contains a three-segmented negative-strand RNA genome, comprising a large (L), medium (M) and small (S) segment [12]. The L segment encodes the viral RNA-dependent RNA-polymerase. The S segment encodes the nucleocapsid (N) protein and a non-structural protein named NSs. The polymerase and the nucleocapsid protein together form the replication machinery of the virus. NSs was identified as the major virulence factor and functions as an antagonist of host innate immunity [13]–[17]. The M segment encodes the two structural glycoproteins Gn and Gc as well as two accessory proteins [18]. The first is a 14-kDa nonstructural protein known as NSm, which was shown to have an anti-apoptotic function [19]. The second is a protein of 78-kD of which the function is yet unresolved.

RVFV can be transmitted by an impressive variety of mosquito species, which are globally prevalent. This explains the fear for future RVF outbreaks in currently unaffected areas, such as Europe and the USA, where both large populations of susceptible livestock and vector species are present. The risks of introduction and subsequent spread of the virus are furthermore increasing by continued globalization and climate change, respectively [20]–[24]. Only recently, a new virus of the *Bunyaviridae* family, the Schmallenberg virus (SBV, genus *Orthobunyavirus*), emerged and spread within a few months accross Europe [25]. SBV was able to spread so rapidly due to the abundance of competent *Culicoïdes* midges and large numbers of immunologically naïve target animals. This incursion, as well as that of the bluetongue virus in Northern Europe in 2006, exemplifies how quickly exotic arboviruses can adapt to new environments. The high impact of RVFV on both animal and human health underlines the need for a safe and effective vaccine that can be used to control RVFV not only in areas of endemicity, but also in currently unaffected areas.

Several vaccines are available for veterinary use in South Africa and some other countries on the African continent. A live-attenuated vaccine, the so-called Smithburn vaccine, elicits solid immunity but is not safe for pregnant animals [26], [27]. An inactivated whole-virus vaccine, on the other hand, is safe to use during all physiological stages, but is expensive to produce and requires booster administrations for optimal protection [28]. Recently, a third vaccine was registered for use in South Africa, named Clone 13. This vaccine was shown to be efficacious and safe in animal studies involving sheep [29] and cattle [30]. However, data on its safety and efficacy in the field have yet to be reported.

An alternative strategy to develop a live-attenuated vaccine resulted in the mutagen-attenuated MP-12 strain [31], [32]. Although effective in livestock [33]–[36], concerns remain about its residual virulence [37], [38]. To attenuate the MP-12 vaccine virus further, derivatives were created that contain deletions in the NSm-coding region and/or the NSs gene. The MP-12ΔNSs was found to be poorly immunogenic, whereas the MP-12ΔNSm vaccine showed promise in sheep [38] and cattle [39] immunogenicity studies. A similar approach was used to develop a ΔNSs/ΔNSm vaccine virus based on the virulent ZH501 human isolate, which appears to be safe and protective in pregnant ewes [40].

Alternative vaccine development strategies that focus on optimal safety have resulted in the development of experimental subunit vaccines, DNA vaccines and vector vaccines, most of which await further evaluation in the target species. These candidate vaccines are described in several comprehensive reviews [11], [41]–[44].

To create a vaccine that optimally combines the efficacy of live vaccines with the safety of inactivated vaccines, we and others previously reported the creation of RVFV replicon particles. These particles contain two of the three viral genome segments, L and S, where the NSs gene of the S segment is exchanged for the gene encoding enhanced green fluorescent protein (eGFP) [45], [46]. A single vaccination with the resulting nonspreading RVFV (NSR) vaccine was shown to induce neutralizing antibodies and to protect lambs from viremia and clinical signs [47]. However, minor amounts of viral RNA were detected in the blood of the vaccinated lambs shortly after challenge infection. We recently found that RVFV can be transmitted vertically in ewes without detection of maternal viremia by our most sensitive qRT-PCR [48]. Although viral RNA was not detected in the blood of these ewes, it was detected in different organs of both ewes and fetuses. This finding suggests that sterile immunity is needed to prevent vertical transmission of the virus. To develop a vaccine that induces sterile immunity after a single vaccination, we decided to improve our NSR vaccine by expressing the major immunogenic protein, Gn, from the NSR small genome segment, resulting in NSR-Gn. In the current study, we demonstrate that expression of Gn from the NSR genome improves Gn-specific humoral and cellular immune responses in mice. A subsequent dose-titration experiment in lambs demonstrated that sterile immunity can be achieved by a single vaccination with the NSR-Gn vaccine.

## Materials and Methods

### Ethics statement

All animal experiments were conducted in accordance with the Dutch Law on Animal Experiments (Wod, ID number BWBR0003081) and approved by the Animal Ethics Committee of the Central Veterinary Institute (Permit Numbers: 2012148, 2012108). To minimize suffering of the animals during our vaccination challenge experiment, lambs were humanely euthanized when they could no longer be stimulated to drink, feed or stand.

### Preparation of the challenge virus

A recombinant RVFV was used as challenge virus. The virus was produced from cDNA as described previously [45] with sequences derived from strain 35/74 [49], thereby generating rec35/74. The titer was determined as 50% tissue culture infective dose (TCID_50_) on baby hamster kidney (BHK) cells, using the Spearman-Kärber algorithm. The virus was handled under biosafety level-3 laboratory conditions in class-III biosafety cabinets.

### Cells and growth conditions

BHK cells were grown in Glasgow minimal essential medium (GMEM; Invitrogen, CA, USA), supplemented with 4% tryptose phosphate broth (Invitrogen), 1% minimum essential medium nonessential amino acids (MEM NEAA, Invitrogen), 1% Penicillin-Streptomycin (Invitrogen) and 5% fetal bovine serum (FCS; Bodinco, The Netherlands). BHK-GnGc cells [45] and derivatives thereof were grown in the above described medium supplemented with 10% FCS and 1 mg/ml Geneticin (G-418; Promega, USA). For clarity, complete medium supplemented with either 5 or 10% FCS is here referred to as GMEM(5) and GMEM(10), respectively. Transfections were performed in Opti-MEM® (GlutaMAX™; Invitrogen), supplemented with 0.2% FCS.

### Plasmids

The plasmid encoding the complete L genome segment and the plasmid encoding the S genome segment in which the NSs gene is replaced by the eGFP gene (pUC57-L and pUC57-S-eGFP, respectively) were described previously [45]. In the current work, the eGFP gene of the pUC57-S-eGFP plasmid was replaced for the Gn gene, starting at the fourth methionine of the M segment open reading frame and ending at the sequence “PIPRHAPIPR”. The nucleotide sequence of the Gn gene was codon optimized for expression in human cells. Plasmid pCAGGS-M encodes the complete glycoprotein precursor of RVFV under control of the CMV immediate enhancer/β-actin (CAG) promoter [50].

### Flow cytometry

Cells were washed with PBS and permeabilized and fixed with Cytofix/Cytoperm solution (BD biosciences, NJ, USA). A monoclonal antibody (mAb) specific for the N protein was used as primary antibody and FITC-conjugated anti-mouse IgG was used as the secondary antibody (Santa Cruz Biotechnologies, USA). Measurements were performed using a CyAn ADP flow cytometer (Beckman & Coulter, USA) and data analysis was performed with Kaluza software version 1.2 (Beckman & Coulter).

### Immunofluorescence

BHK cells were infected with NSR-Gn at a multiplicity of infection (MOI) of 0.5 in 24-well plates. Control BHK cells and Rep-Gn cells were seeded as well. After 48 hours of incubation at 37°C and 5% CO_2_, cell monolayers were washed with PBS Ca/Mg and fixed with 4% (w/v) paraformaldehyde for 15 min. The cells were washed three times with PBS and permeabilized, when required, with 1% Triton for 5 min. Cells were washed three times with washing buffer (PBS, 0.05% v/v Tween-20) followed by a 30 min incubation in washing buffer containing 5% FCS. Cells were incubated with mAb 4-39-cc, which specifically recognizes Gn [51], for 1 h at 37°C. The cells were subsequently washed three times with washing buffer and incubated with Texas Red-conjugated anti-mouse IgG2b (Beckman & Coulter). Cell nuclei were stained with DAPI staining (Invitrogen), according to the manufacturers' instructions. Images were taken with an AMG EVOSfl fluorescent microscope.

### Preparation of the vaccine

To produce replicon particles containing the S genome segment encoding either eGFP (S-eGFP) or Gn (S-Gn), cells of the corresponding replicon cell lines were seeded in T150 cell culture flasks at a density of 7×10^6^ cells/flask in GMEM(10). Medium was exchanged the next day for 18 ml Opti-MEM and the cells were transfected with a mixture of 14 µg of the pCAGGS-M plasmid DNA and 40 µl JetPEI transfection reagent (Polyplus-transfection SA, France) in 2000 µl saline, following the manufacturers' instructions. Culture medium was harvested the day after transfection and cleared from cell debris by centrifugation at 4500 x*g* for 15 min. When required, replicon particles were concentrated by ultra-centrifugation at 64 000 *xg* for 2.5 h and resuspended in GMEM(5). NSR-Gn replicon particles to be used for the vaccination of sheep were diluted in Opti-MEM, yielding a high dose (10^6.3^ TCID_50_/ml), a medium dose (10^4.6^ TCID_50_/ml) and a low dose (10^4.0^ TCID_50_/ml). The indicated NSR-Gn titers were determined after vaccination.

### Vaccination of mice

Six-week-old female BALB/cAnCrl mice (Charles River Laboratories) were housed in two groups of 6 animals and one group of 4 animals and were kept in type III filter top cages under BSL-3 conditions. Mice were allowed to acclimatize for 6 days, after which the groups of 6 animals were vaccinated with a titer of 5×10^5^ TCID_50_ of either the NSR vaccine or the NSR-Gn vaccine. The group of four mice was mock vaccinated with GMEM(5). Vaccines were administered in a volume of 50 µl by injection into the thigh muscle using a 25 gauge, 16 mm needle. The mice were observed daily and no signs of illness were recorded. Blood samples were obtained on -1, 13, 22 and 29 days post vaccination (DPV). On DPV 29, the mice were euthanized by cervical dislocation and spleens were collected.

### Enzyme-linked immunospot (ELISPOT) assay

A mouse interferon (IFN)-γ antibody pair, consisting of a capture and a detection antibody (Becton Dickinson, USA) was used to determine the number of IFN-γ secreting spleen cells. MultiScreen_HTS_ (Millipore, USA) plates were coated overnight with the capture antibody at 4°C. Unbound antibody was removed by washing with PBS. The plates were then blocked with cRPMI (RPMI 1640, Invitrogen, supplemented with 10% FCS, 1% L-glutamine, 0.1% BME and 1% penicillin/streptomycin) for 30 min at room temperature (RT). Spleens were collected from mice immediately after euthanization and spleen cells were isolated as described [52]. Briefly, spleens were gently crushed in a 70 µm cell strainer (BD Bioscience) and cells were collected by washing the strainer with cRPMI. Erythrocytes were depleted by incubation of the cells in 5 ml ACK cell lysis buffer (Invitrogen) for 5 minutes. Cells were washed with cRPMI and counted with a Scepter cell counter (Millipore), supplied with a 40 µm sensor. The splenocytes were seeded at a density of 5×10^5^ cells/well in cRPMI in triplicate and stimulated overnight at 37°C and 5% CO_2_ with a peptide derived from the Gn protein: SYAHHRTLL [53], a peptide derived from GFP: HYLSTQSAL [54] or a peptide derived from the nucleoprotein of influenza virus: TYQRTRALV [55]. Peptides were synthesized by the Genscript Corporation (USA) and used at a concentration of 5 µg/ml. Additionally, the ectodomain of the Gn glycoprotein [56] was used for stimulation at a concentration of 10 µg/ml. Concanavalin A (1 µg/ml) was used as a positive control and cRPMI as a negative control. After 10 h of stimulation, the cells were removed with chilled water and the plates were extensively washed with washing buffer (PBS, 0.05% v/v Tween-20). Detection of spots was performed by incubation of the plates with biotinylated detection antibodies for 2 h at room temperature, followed by 3 washing steps and an incubation with alkaline phosphatase (ALP)-conjugated streptavidin (Mabtech, Sweden) for 1 h at RT. Plates were washed 4 times with washing buffer and 3 times with PBS to remove residual Tween-20. Spots were developed with BCIP/NBT chromagen (Thermo scientific, USA) for 20 min. Plates were dried in a dry incubator at 37°C. Spots were counted with a CTL ImmunoSpot apparatus. The number of specific spots was determined as the average of three repetitions from each sample after the average number of the respective negative controls was subtracted.

### Virus neutralization test with mouse sera

Virus neutralization tests (VNT) with mouse sera were performed as previously described [45], with some modifications. Briefly, sera were diluted 1∶10 in a 96-well plate and then serially diluted (2-fold) in a volume of 25 µl in GMEM(5). Another 25 µl of GMEM(5), containing ∼50 NSR particles was added to each serum dilution and incubated for 1.5 h at 37°C. Next, 30, 000 BHK cells in 50 µl of GMEM(5) were added to each well. After 36–48 hours, the NSR neutralization was determined and titers were calculated using the Spearman-Kärber method [57], [58]. All samples were tested in triplicate.

### Vaccination and challenge of lambs

Texel crossbreed lambs were divided into four groups of eight animals. At 9–10 weeks of age, lambs were vaccinated via the intramuscular route (right thigh) with a high-dose (HD, 10^6.3^ TCID_50_/ml), medium dose (MD, 10^4.6^ TCID_50_/ml) or low dose (LD, 10^4.0^ TCID_50_/ml) of the NSR-Gn vaccine in a 1 ml volume or mock-vaccinated with Opti-MEM. Three weeks after vaccination, all lambs were challenged via the intravenous route with 10^5^ TCID_50_ of RVFV rec35/74. Animals were sedated before intravenous inoculation (jugular vein) by intramuscular administration of medetomidine (40 µg/kg medetomidine hydrochloride, Sedator®, Eurovet, The Netherlands). For vaccination and challenge, 18 gauge, 25 mm needles were used. Rectal temperatures were determined daily from the day of arrival until the day of euthanasia. Fever was defined as a rectal temperature above 40.5°C. This threshold was determined as the average plus three times the standard deviation of the rectal temperatures of all animals, measured from −7 to −1 DPC. EDTA blood samples were obtained daily during the first week following challenge and subsequently on 9, 11, 14 and 21 days post challenge (DPC). Serum samples were collected on −21, −14, −7, 0, 7, 14 and 21 DPC. The surviving animals were euthanized three weeks after the challenge by exsanguination, after being anesthetized with 50 mg/kg sodium pentobarbital (Euthasol®, ASTfarma BV, The Netherlands) applied via the intravenous route. Plasma samples were analysed with quantitative real-time PCR as described previously [47]. Virus isolation was performed as previously described [47]. Sera were analysed for the presence of anti-N antibodies with a commercial competition ELISA (ID-VET, France) according to the manufacturers' instructions. Virus neutralization titers were determined by a virus neutralization test (VNT) as described [59].

### Statistical analysis

Data from the ELISPOT assay and the virus neutralization assay of the mouse experiment were analysed with the Mann-Whitney non-parametric test, using GraphPad Prism version 5.00 for Windows (GraphPad Software, USA). Statistical differences with *p*-values<0.05 were considered significant.

## Results

### Creation of a replicon cell line expressing Gn

**Figure 1:**
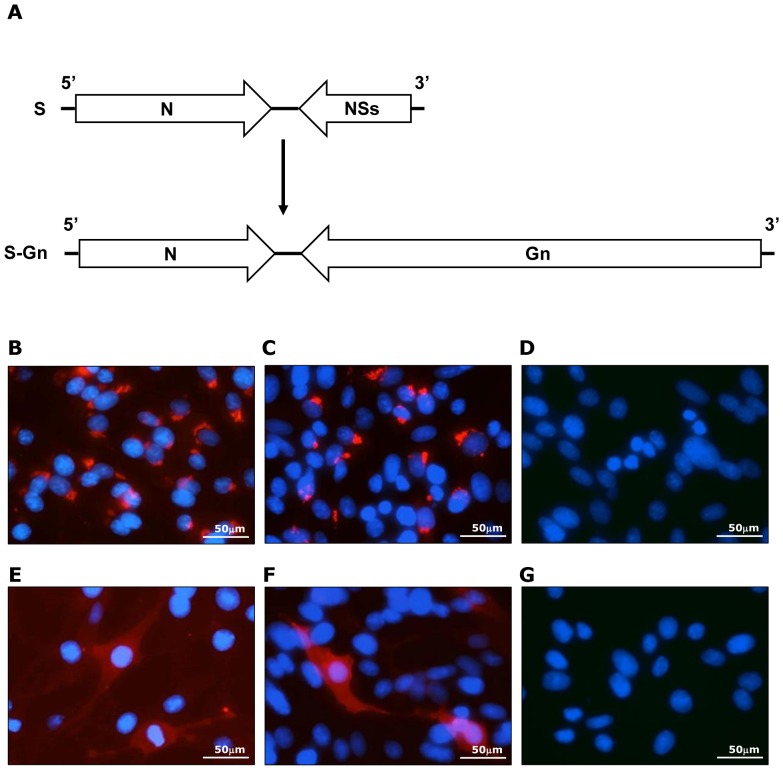
Construction of the S-Gn segment and expression of Gn. (A) Schematic representation of the S segment of RVFV (upper panel) and the S segment in which the NSs gene is replaced for the codon-optimized Gn gene (lower panel). Distribution of Gn in Rep-Gn cells (B and E) and in BHK cells infected with NSR-Gn at an MOI of 0.5 (C and F). Panels D and G represent BHK control cells. Upper panels represent permeabilized cells and lower panels represent nonpermeabilized cells. The cells were stained with an anti-Gn monoclonal antibody and a Texas Red-labeled secondary antibody. Nuclei were visualized by DAPI staining.

To produce replicon particles that express the Gn glycoprotein from the S genome segment, the NSs gene was replaced for a codon-optimized Gn gene (Fig. 1A). A cell line that constitutively maintains the resulting S-Gn genome segment together with the L genome segment was created essentially as described previously [45]. Briefly, BHK-GnGc cells were infected with fowlpox virus expressing T7 polymerase and subsequently transfected with the pUC57 plasmids encoding the L and S-Gn genome segments, together with plasmid pCAGGS-M, which encodes the glycoprotein precursor. Cells were repeatedly passaged and transfected after every cell passage with pCAGGS-M, until more than 98% of the cells became positive for N protein expression, as determined by flow cytometry. The resulting cell line was named Rep-Gn.

The expression and localization of the Gn protein in the Rep-Gn cells was analyzed by immunofluorescence. Consistent with previous reports [60], [61], in permeabilized cells, Gn was mainly distributed perinuclearly, corresponding to Golgi localization (Fig. 1B). In cells with intact cell membranes, Gn was detected at the cell surface (Fig. 1E). Transfection of the Rep-Gn cells with the pCAGGS-M glycoprotein expression plasmid resulted in the production of replicon particles that contain the L and S-Gn segment. The yield of the NSR-Gn particles was determined at 10^7^ TCID_50_/ml. These particles were used to infect BHK cells and the synthesis of the Gn protein was analyzed by immunofluorescence. The *de novo* synthesized protein showed a similar distribution as observed in the Rep-Gn cell line (Fig. 1C and F).

### Immune responses elicited by NSR and NSR-Gn vaccination of mice

**Figure 2:**
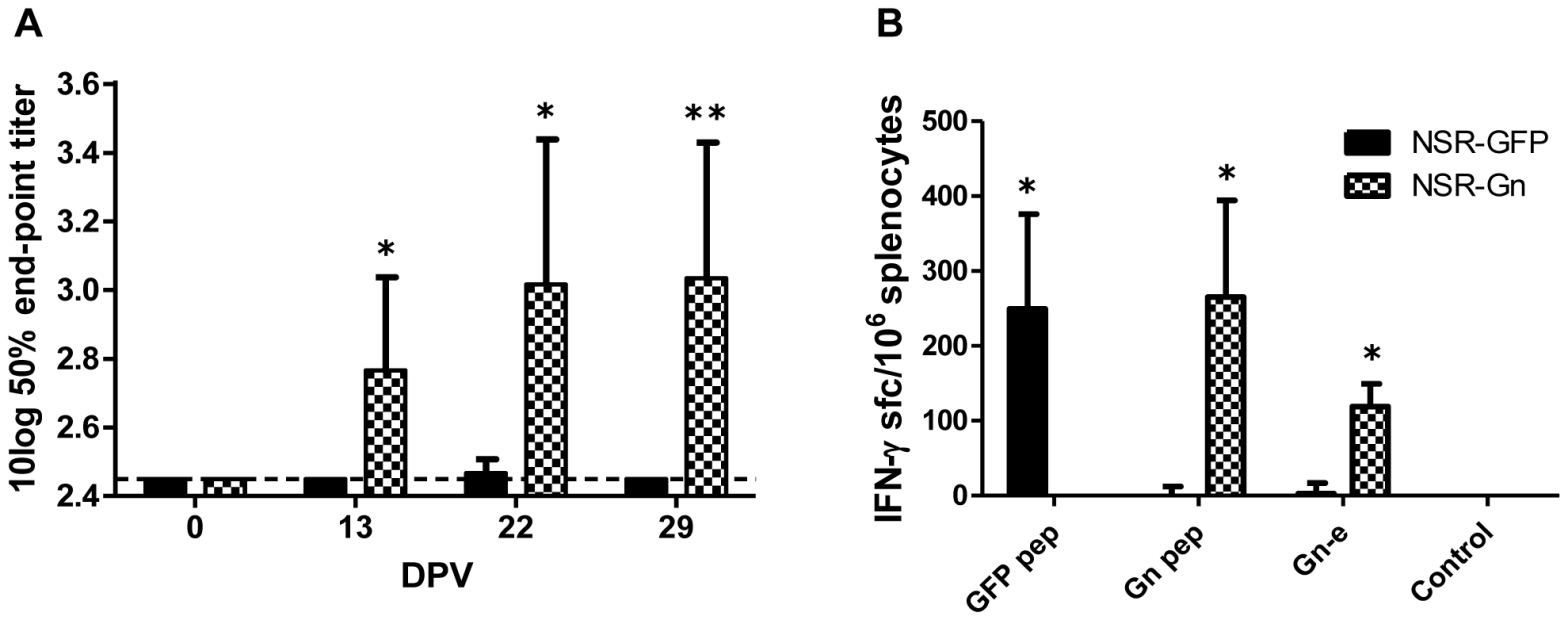
Humoral and cellular immune responses elicited by vaccination of mice with NSR or NSR-Gn. (A) VNT titers in sera collected from mice vaccinated with NSR or NSR-Gn before vaccination (DPV -1) and 13, 22 and 29 DPV. Bars represent average titers (n = 6) of each group with standard deviation. The detection limit of the assay is depicted by the interrupted line. (B) Detection of IFN-γ-producing splenocytes isolated from mice vaccinated with NSR or NSR-Gn. Splenocytes were isolated and seeded at a density of 5×10^5^ cells/well in triplicate and stimulated for 12 hours with the indicated peptides or the ectodomain of Gn. Bars represent an average number of IFN-γ producing cells (n = 4) per group with standard deviation. The non-parametric Mann-Whitney test was used for statistical analysis and statistical significance between the groups is depicted by asterisks (*p<0.05; **p<0.01).

Groups of six mice were vaccinated once via the intramuscular route with either NSR or NSR-Gn, and four mice were mock-vaccinated. Sera were collected the day before vaccination and on DPV 13, 22 and 29. Virus neutralization tests (VNT) revealed the presence of neutralizing antibodies in the sera of the NSR-Gn-vaccinated animals on DPV 13, and the titers increased until DPV 22, after which they remained unchanged until 29 DPV (Fig. 2A). The VNT titers of the NSR vaccinated group were marginal. No titers were detected in the mock vaccinated group (data not shown).

Spleens were collected on DPV 29 from four mice of each group and from three control mice. Splenocytes were isolated and stimulated with peptides representing previously reported CD8-restricted epitopes of the GFP protein or the RVFV Gn protein. An unrelated peptide derived from the nucleoprotein of influenza virus was used as a negative control. Additionally, the ectodomain of the Gn protein was used for cell stimulation to estimate predominantly the Gn-specific CD4 response. The GFP peptide induced a clear response in splenocytes from all four NSR-vaccinated mice. Analyses with the Gn peptide or the Gn ectodomain revealed significantly higher numbers of IFN-γ producing cells in NSR-Gn-vaccinated mice than in those vaccinated with NSR. The control peptide did not induce a measurable response in splenocytes of any of the mice (Fig. 2B). None of the stimulating agents induced IFN-γ secretion in the spleen cells of the mock vaccinated mice (data not shown).

### Sheep dose-titration study

**Figure 3:**
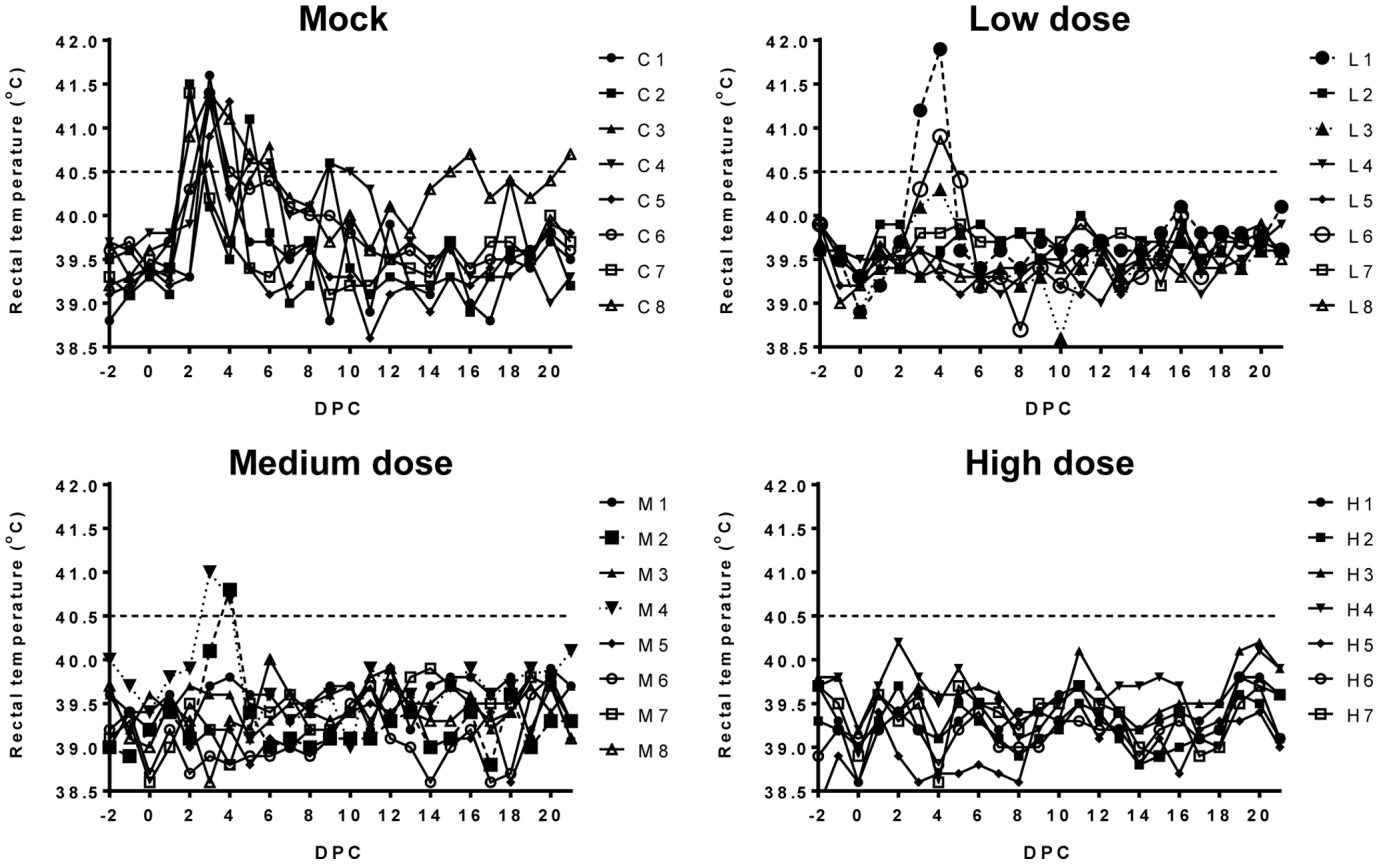
Rectal temperatures of vaccinated and mock-vaccinated lambs before and after challenge with RVFV. Fever was defined as a rectal body temperature above 40.5°C (interrupted line). Body temperatures of mock-vaccinated lambs (C1–C8) and lambs vaccinated with a low dose (L1–L8), medium dose (M1–M8) or high dose (H1–H7) of NSR-Gn are depicted individually.

After establishing that expression of Gn from the small genome segment of NSR particles contributes to both cellular and humoral immunity, we proceeded with a sheep study to determine the minimum protective dose. Groups of 8 lambs were vaccinated with either 10^4^ TCID_50_/ml (low dose), 10^4.6^ TCID_50_/ml (medium dose) or 10^6.3^ TCID_50_/ml (high dose) of NSR-Gn. One group of 8 lambs was included as a challenge control (mock) group. All lambs were challenged at 21 DPV. Consistent with our previous experiments, mock-vaccinated lambs developed fever within 2 days after challenge, which lasted on average for four days. In four lambs of the mock group the fever was multiphasic (Fig. 3). One lamb in this group (C3) died 7 days after challenge. Post mortem examination revealed findings typical for RVF and pneumonia. Two lambs of the low-dose group and two lambs in the medium-dose group developed fever. When fever occurred in vaccinated animals, it was delayed with one day and no secondary peaks were observed. None of the lambs from the high-dose group developed fever. One of the lambs in this group died from esophagus obstruction two days after the vaccination, therefore data from only seven animals are shown.

**Figure 4:**
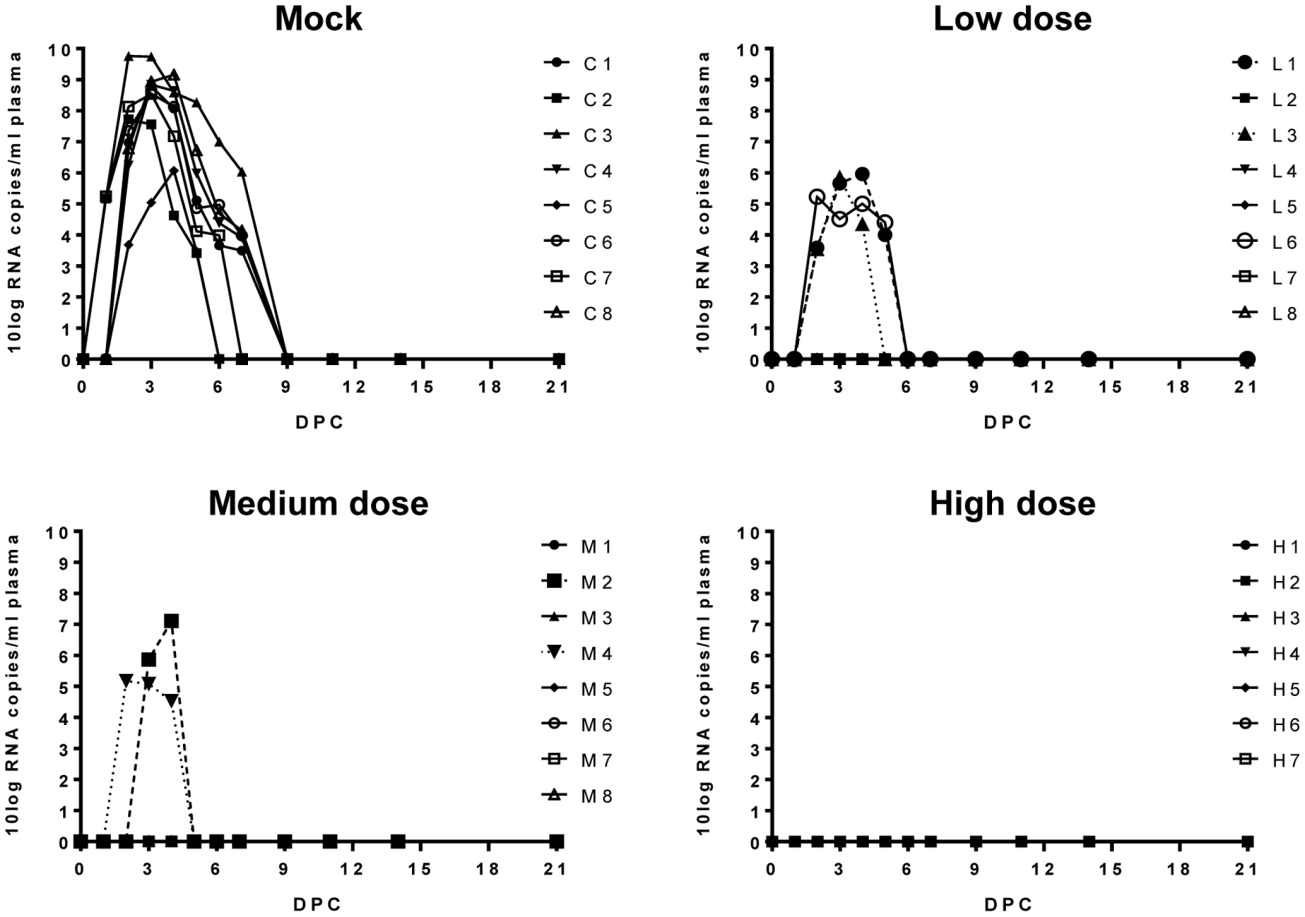
Detection of viral RNA in plasma by qRT-PCR. Plasma samples were collected daily at the first 7 days post challenge (DPC) and subsequently on DPC 9, 11, 14 and 21. Viral RNA copy numbers detected in individual animals of the mock-vaccinated group (C1–C8), low-dose group (L1–L8), medium-dose group (M1–M8) and high-dose group (H1–H7) are depicted.

All mock-vaccinated lambs developed high viremia, reaching maximum values between 10^6^ and 10^9.8^ RNA copies/ml plasma as detected by PCR. Viremia lasted for 4 to 7 days (Fig. 4). In the low-dose and medium-dose groups, viremia correlated with fever but was of shorter duration compared to the mock group. No viremia was detected in lambs vaccinated with the high dose of NSR-Gn.

The plasma samples with the highest PCR titers from each animal were subjected to virus isolation. Only samples with PCR titers above 10^5.5^ RNA copies/ml were used as previous attempts to isolate virus from samples with lower PCR titers were always unsuccessful. Virus was successfully isolated from the plasma of all mock vaccinated animals and from two (L1, L3) and one (M2) plasma samples of the low-dose and medium-dose vaccination groups, respectively (data not shown).

Livers and brains of all animals were tested for the presence of viral RNA with qRT-PCR at the end of the experiment. In the mock vaccinated group, viral RNA was found in the livers of three lambs (C3, C4 and C6) and in the brain of one lamb (C8). None of the vaccinated animals was found positive for viral RNA in the liver or brain.

### Antibody responses in sheep

**Figure 5:**
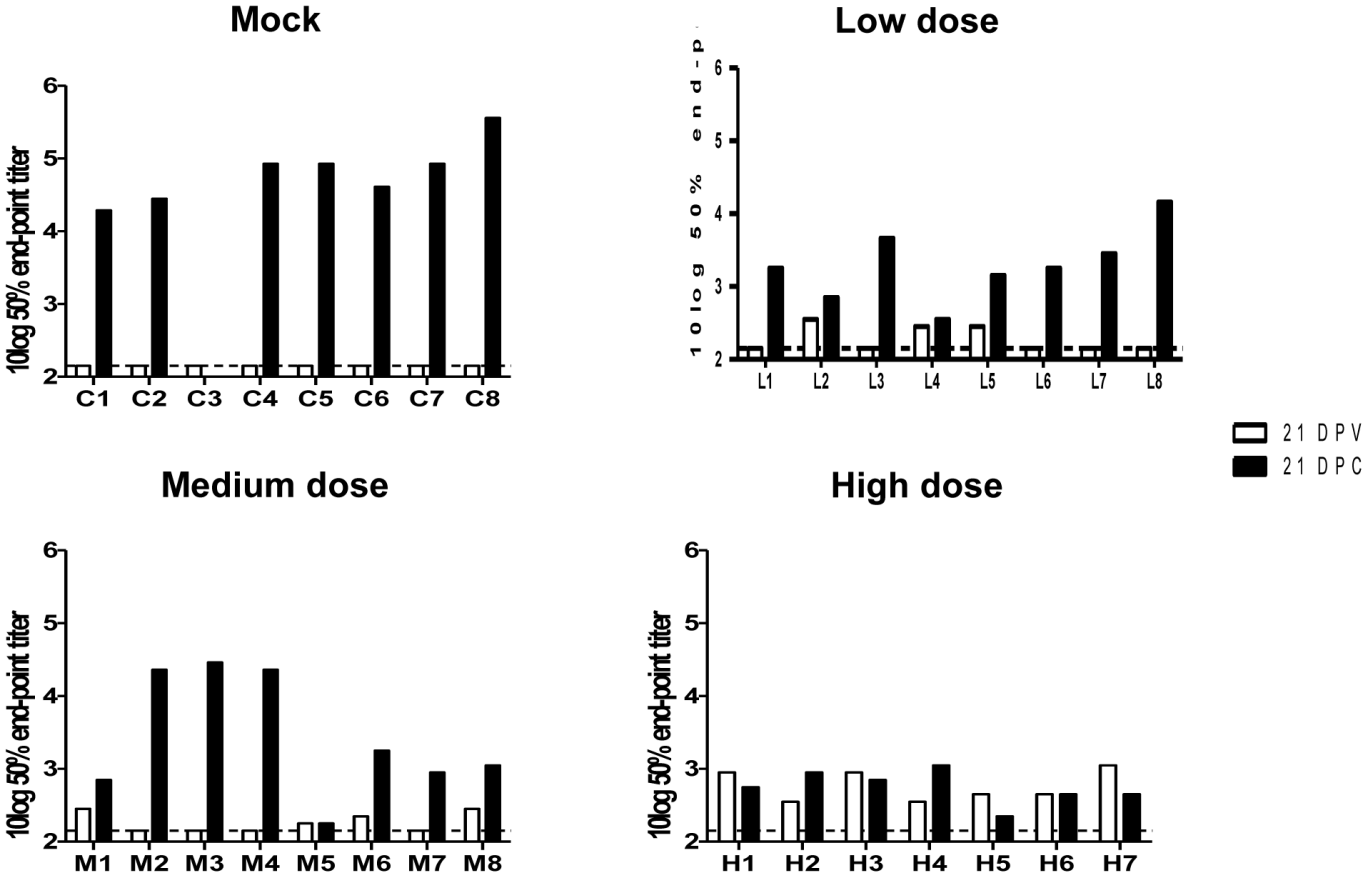
Virus neutralization test (VNT). Sera were obtained from lambs of the mock group, low-dose group, medium-dose group and high-dose group. The white bars represent VNT titers determined 21 days post vaccination (DPV) and the black bars represent the VNT titers determined 21 days post challenge (DPC). Results obtained from analysis of each individual animal from the mock-group (C1–C8), low-dose group (L1–L8), medium-dose group (M1–M8) and high-dose group (H1–H7) are depicted. The detection limit of the assay is represented by an interrupted line. Lamb C3 died 7 days after challenge, therefore no serum sample was collected at 21 DPC.

Sera collected from all animals on the day of vaccination, on the day of challenge, and three weeks after challenge were tested for virus neutralizing activity. Neutralizing antibodies were not detected in any of the sera collected on the day of vaccination (data not shown) and no neutralizing antibodies were detected in the sera of the mock-vaccinated animals collected three weeks after vaccination. Three animals vaccinated with a low dose of NSR-Gn and four animals that received a medium dose developed neutralizing antibodies after vaccination (Fig. 5). Lambs in these groups generally displayed an increase in the neutralizing antibody titers after challenge, being more prominent in animals that did not have neutralizing antibodies before challenge. All animals that received a high dose of NSR-Gn developed neutralizing antibodies and antibody levels were highest in this group before the challenge infection. These neutralizing antibody levels did not increase after challenge. The animals of the mock group developed the highest VNT titers upon challenge.

**Figure 6:**
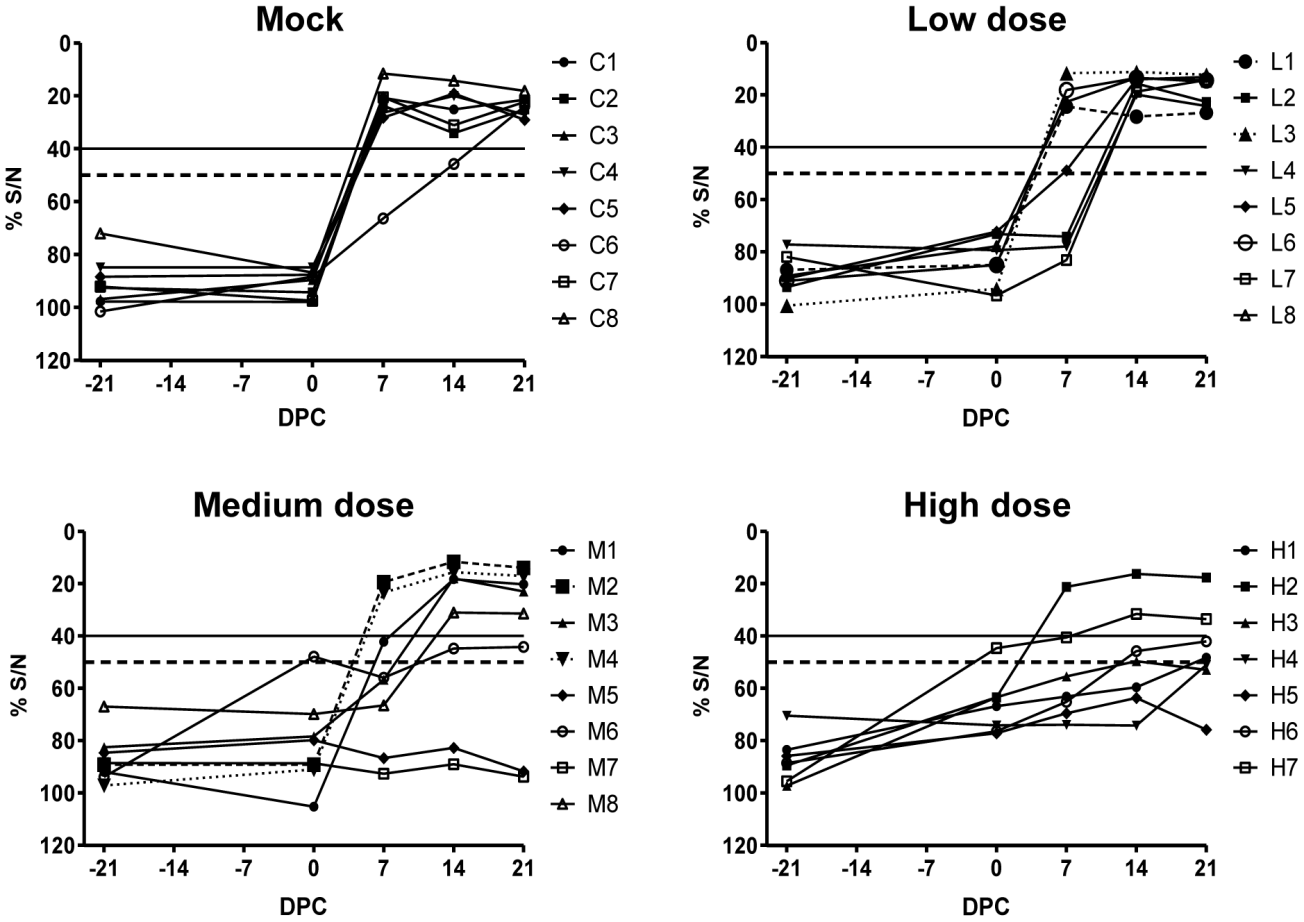
Detection of anti-N antibodies by ELISA. Sera were obtained 7, 14 and 21 days post vaccination (DPV) and at 0, 7, 14 and 21 days post challenge (DPC). Titers are expressed as percentage competition ratio of the optical densities (OD) of the sample and the OD of the negative control (%S/N). All values lower than 40% are considered positive, between 40–50% are considered doubtful and above 50% are considered negative. The 40% and 50% cut-offs are represented by solid and interrupted lines, respectively. Results obtained from analysis of each individual animal from the mock-group (C1–C8), low-dose group (L1–L8), medium-dose group (M1–M8) or high-dose group (H1–H7) are depicted.

In addition to determining the VNT titers, sera were analyzed for the presence of antibodies against the N protein using the commercial ID Screen® Rift Valley fever Competition ELISA kit (ID-Vet, Montpellier, France). Seven of the eight sera from the mock-vaccinated animals were scored positive already on day 7 after challenge and in one animal a delayed increase in the antibody response was observed (Fig. 6). In the low-dose group, four animals developed an anti-N response similar to that observed in challenged mock-vaccinated animals and in four other animals the antibody response was delayed by 7 days (Fig. 6). By DPC 21, all sera were found positive for anti-N antibodies. Five sera of the lambs vaccinated with the medium dose were found positive for anti-N antibodies, one was doubtful and in two animals no anti-N antibodies were detected at 21 DPC. In the high-dose group, two samples were found positive, two were deemed doubtful and three were found negative for the presence of anti-N antibodies at 21 DPC. Although the serum samples from this group were scored negative before the challenge, there was a clear increase in the %S/N values, as compared to the mock group and the low- and medium-dose groups.

## Discussion

Vaccines based on replicons can optimally combine the efficacy of live vaccines with the safety of inactivated or subunit vaccines. Vaccination with the recently developed NSR replicon vaccine was shown to reduce challenge virus viremia in lambs to levels undetectable by virus isolation. Low levels of viral RNA were, however, detected in the blood of challenged animals by a highly sensitive qRT-PCR. We recently found that RVFV can be transferred to the fetus in gestating ewes even when viremia is not detectable by qRT-PCR. We therefore aimed to improve our NSR vaccine so that sterile immunity can be achieved. To this end, the Gn glycoprotein, which is the major target for neutralizing antibodies, was introduced in the NSR genome.

Memory T-cell responses were analyzed using spleen cells of mice that were vaccinated with NSR expressing eGFP (NSR) or NSR expressing Gn (NSR-Gn). Stimulation of splenocytes with a GFP-derived peptide resulted in IFN-γ production by spleen cells of the NSR-vaccinated group. As the eGFP protein is not present in the NSR particle, we conclude that the observed response results from eGFP production *in vivo*. Importantly, a Gn-derived peptide stimulated significantly higher numbers of IFN-γ producing splenocytes in cultures obtained from NSR-Gn-vaccinated mice, compared to cultures derived from NSR-vaccinated mice. Although the Gn protein is incorporated in the NSR particle, expression of the Gn gene from the NSR genome resulted in a superior and sustained memory CD8 response. Soluble ectodomain of Gn (Gne) was also used for splenocyte stimulation. Soluble proteins are internalized by antigen presenting cells by endocytosis and are presented mainly by MHC-II [62], [63]. The observed stimulation by Gne therefore likely represents a CD4^+^ T-cell response. Once again, splenocytes collected from NSR-Gn-vaccinated animals displayed the strongest response, indicating that the *in vivo* expression of Gn contributes substantially to the immunogenicity of the NSR-Gn vaccine. In addition, the VNT titers of NSR-Gn-vaccinated animals were readily detectable by 13 DPV, while the titers of the NSR group were marginal. This result demonstrates that expression of Gn by NSR-Gn not only promotes priming of IFN-γ secreting T-cells, but also stimulates neutralizing antibody responses. The combined results of this experiment underscore the added value of Gn expression from the NSR genome.

We previously demonstrated that a single vaccination with 10^7^ TCID_50_ of NSR protects lambs from viremia and clinical signs, although protection was not sterile [47]. Since we anticipated superior efficacy of the NSR-Gn vaccine, we continued with determining the minimal protective dose of this vaccine in lambs. Vaccination with a low dose (10^4.0^ TCID_50_/ml) prevented detectable viremia in five lambs and strongly reduced viremia in the remaining three animals. No viremia was detected in 6 of 8 lambs that were vaccinated with a medium dose (10^4.6^ TCID_50_/ml), whereas two lambs did develop low-level viremia. It is important to note, however, that neutralizing antibodies were boosted after challenge in these two groups. A sharp increase in the anti-N response was noted in the low-dose group after challenge, similar to that observed in the mock group. The group of lambs vaccinated with a medium dose displayed a more variable pattern, with two animals not developing anti-N antibodies and two others showing only a minor increase in the anti-N responses after challenge. The observed boosts of anti-N antibodies as well as neutralizing antibodies after challenge suggests that virus replication occurred at a low level in the lambs of these two groups. Some lambs of the low-dose and medium-dose groups did not develop detectable levels of neutralizing antibodies after vaccination, but were nevertheless solidly protected from challenge infection. The observed protection in these animals is therefore attributed to T-cell responses. No viral RNA was detected upon challenge in any of the lambs vaccinated with the high dose (10^6.3^ TCID_50_/ml) of NSR-Gn. This result correlates with the absence of an anamnestic antibody response after challenge. Taken together, these data suggest that challenge virus was not able to replicate in these lambs and that sterile immunity was achieved.

Although a clear dose-response was demonstrated in this study, it is interesting to note that even vaccination with a low dose of NSR-Gn can provide sterile immunity in lambs. After inoculation of NSR-Gn in the muscle, the nonspreading virus can infect not only muscle cells, but also macrophages and dendritic cells. RVFV was recently shown to use dendritic cell (DC)-specific intercellular adhesion molecule 3-grabbing nonintegrin (DC-SIGN) as a receptor, which is present on both dendritic cells and macrophages [64]. DCs and macrophages are possibly used by RVFV as “trojan horses” to migrate to secondary sites of replication, such as the lymph nodes. While doing so, the virus must prevent the activation of these immune cells, in which the NSs protein is likely to play a role. Since NSs is lacking from NSR-Gn particles, these particles are probably unable to prevent activation of the macrophages and DCs, thereby facilitating a potent priming of the immune response. We propose that vaccination with a low dose of NSR-Gn can provide sterile immunity if a sufficient number of macrophages and dendritic cells are infected by NSR-Gn. If this hypothesis proves to be correct, it will be interesting to target dermal dendritic cells by intradermal vaccination with the goal to lower the protective dose of the NSR-Gn vaccine.

In conclusion, here we demonstrate that expression of the Gn protein from the NSR genome results in improved cellular and humoral immune responses and that the resulting NSR-Gn vaccine provides sterile immunity in lambs after a single vaccination. Given its high efficacy and safety profile, it would be valuable to evaluate the use of the NSR-Gn vaccine as a human vaccine as well.
